# The necessary, albeit belated, transition to computerized cognitive assessment

**DOI:** 10.3389/fpsyg.2023.1160554

**Published:** 2023-04-24

**Authors:** David Asensio, Jon Andoni Duñabeitia

**Affiliations:** ^1^Facultad de Lenguas y Educación, Universidad Nebrija, Madrid, Spain; ^2^AcqVA Aurora Center, UiT The Arctic University of Norway, Tromsø, Norway

**Keywords:** cognitive assessment, cognition, digital tools, computerized cognitive assessment, paper and pencil test

## Abstract

Cognitive assessment is a common and daily process in educational, clinical, or research settings, among others. Currently, most professionals use classic pencil-and-paper screenings, tests, and assessment batteries. However, as the SARS-CoV-2 health crisis has shown, the pencil-and-paper format is becoming increasingly outdated and it is necessary to transition to new technologies, using computerized cognitive assessments (CCA). This article discusses the advantages, disadvantages, and implications of this necessary transition that professionals should face in the immediate future, and encourages careful adoption of this change to ensure a smooth transition.

## 1. Introduction: the current state of cognitive assessment

Cognitive assessment refers to a set of techniques and procedures to ascertain the status of one or more aspects of a person’s cognitive profile. It is an essential process that is performed daily in clinical, academic, and research settings around the world. It is often used to identify behavioral markers of processes involving cognitive impairment (Wild et al., 2008) or neurodegeneration (Choi, 2008; Davis et al., 2015; Daroische et al., 2021), although it is also used in healthy users (White et al., 2012; Bertelli et al., 2018). These tests can take many forms, consist of different activities, have different application rules, and require different types of answers from the user. However, to a greater or lesser extent, they can all be applied in different formats, the main versions being paper-and-pencil and computerized.

Mainly for reasons of tradition and accessibility, most of the widely applied tests are paper-and-pencil tests. Classical examples include the Montreal Cognitive Assessment (MoCA; Nasreddine et al., 2005), the Stroop Test (Stroop, 1935), Raven’s progressive matrices (RSPM; Raven, 1938), Rey Auditory Verbal Learning Test (RAVLT; Rey, 1941; Schmidt, 1996), Trail Making Test (TMT; Reitan, 1955), the Corsi Block-Tapping Test (Corsi, 1972), the mini-mental state examination (MMSE; Folstein et al., 1975), Wisconsin Card Sorting Test (WCST; Heaton, 1981), Boston Naming Test (Kaplan et al., 1983), and the Wechsler scales (Wechsler, 2008; Wechsler, 2014). While quite a few of these individual tests have been digitized (Hannukkala et al., 2020), most of the assessment batteries that usually contain them have not finished making the step into the digital modality, only timidly entering the field through digitized corrections, without coming fully online. In addition, it should be noted that there is a relatively wide range of variability in the digitalization feasibility of the tests. On the one hand, the translation to digital format is straightforward for tests that require selecting one or more answers from an array of options on paper. On the other hand, tests that are manipulative or visuo-constructive, or that require drawing or complex verbal responses, represent a challenge or imply complex adaptation processes for their implementation on digital devices.

In contrast, more recently developed tests and assessment batteries are now being created fully digitized, to the point that some of them have not even been distributed in physical format. These are generally called computerized cognitive assessments (CCA), and some of the most noteworthy examples include the CogniFit General Cognitive Assessment Battery (CAB)^™^ (Haimov et al., 2008), or the Cambridge Neuropsychological Test Automated Battery (Sandberg, 2011), in addition to those that Zygouris and Tsolaki (2014) already pointed out in their review. This article outlines the advantages, disadvantages and implications of this transition to digital.

## 2. Reasons for the transition

Before the 2000s, it did not make sense to use digitized tests, because of the cumbersome, expensive and infrequent equipment required. However, nowadays children are educated in the use of technology (Lee et al., 2014; Wojcik et al., 2022), adults work with it every day, and older people are gaining experience and confidence in the use of these devices (Demiris et al., 2004; Kim and Preis, 2015; Kim et al., 2016). Thus, technological advances and easier access to technology have made CCAs feasible for everyday use.

The transition to CCAs is supported by different cognitive theories and psychometric approaches. For instance, computer-based assessments allow customizing the tests to the test-takers along the principles of the Item Response Theory (Mead and Drasgow, 1993; Huebner, 2010). Besides, the Cognitive Load Theory (Sweller, 1988) suggests that the computer-based format decreases ineffective cognitive load with respect to the paper-based format, thus optimizing performance (Khalil et al., 2010; for further discussion, see Taylor 1994).

Given this paradigm shift, it seems reasonable to foster a transition to the coexistence between classic cognitive assessment tools and CCA to always offer the option that best fits the circumstances of each evaluation session, evaluator, and person being assessed. The greatest example of the need to implement CCA as a regular tool and that classical tests can become non-operational has been unintendedly offered by the lockdown resulting from the SARS-CoV-2 health crisis (Larner, 2021). Many health and education professionals, and researchers who needed to assess the cognitive status of their informants have had to either pause their activity or migrate to new technologies because the classic cognitive assessment tools they used to apply were no longer suited to the needs of the situation. In fact, these circumstances have changed the modality of assessment, but not the frequency with which they are used (Webb et al., 2021). With this in mind, a selected literature review of the status and needs in the field of cognitive assessment has been conducted, and the pros and cons of transitioning to CCAs have been analyzed.

## 3. The advantages of CCA

Numerous studies address the advantages of computerized cognitive assessments over classic pencil-and-paper tests (Sternin et al., 2019). The most basic advantage is familiarity with the delivering device since digital technology is deeply integrated into our lives. Most people not only make use of computers, tablets, and smartphones daily, but also master these devices.

Digital platforms are also well-known for their accessibility, and the possibility of modifying the contrast, the size or color of the text, the volume, or the response requirements of people with reduced mobility or motor difficulties represent altogether a clear-cut advantage of CCA. The adaptation of the test to the specific needs of the assessed person is straightforward in CCA, including language modification when it is necessary to serve a student, patient, or user of another language (see Frances et al., 2020, for a discussion of the impact of this factor). Since COVID-19, ubiquity takes an important role, and the need to remotely assess people has skyrocketed (Larner, 2021). In this line, several studies point to the feasibility of remote cognitive assessments (Settle et al., 2015; Geddes et al., 2020; Zeghari et al., 2021).

Usability and user experience can also be taken as a clear advantage of CCA over traditional assessments. Interactive activities, automatic visual and auditory feedback, and colorful stimuli result in more engaging, motivating, and user-friendly assessments for the test-taker and are easier for the evaluator (Soto-Ruiz, 2020). But importantly, the CCA does not have to lose psychometric value because of their customizable digitized format. In fact, psychometric characteristics such as discriminant validity or test–retest reliability of CCAs are usually well documented, as in the case of conventional paper-and-pencil resources (Zygouris and Tsolaki, 2014). In fact, the ability of the device itself to record reaction times, to be extremely accurate in identifying user responses, to provide the same feedback to all users equally, to provide instant results, or to support the incorporation of more sophisticated techniques such as eye movement detection, are some of the major advantages of CCA.

Another advantage that may not be as obvious, but that stands as a critical one, is the updateable nature of the scales. If test distributors regularly update the reference scales of test scores, there is no need to buy new versions of the test to stop comparing test takers with outdated scales. This is a relatively natural process for the CCA given that the update may take place in a server, without imposing any burden on the evaluator. In contrast, any update in the reference norms of a paper-and-pencil may require renewal actions on the side of the evaluator.

## 4. In the way to transition

What keeps professionals attached to cognitive analog assessments? The CCA have some limitations that cannot be ignored and that will make it preferable on certain occasions to use analog tools instead of digital ones. There are still people who are digital illiterates or that have a low technological-digital command, and they may manage well or better with paper and pencil. This may be particularly important in certain pathologies, and as Witt et al. (2013) pointed out, the CCA cannot completely replace a comprehensive paper-and-pencil neuropsychological assessment in certain circumstances.

However, one of the main problems that prevents professionals from making the transition is more contextual and cultural. On the one hand, professionals are habituated to using analog screenings, tests, and cognitive assessment batteries. They have been historically trained with them, they have applied them hundreds of times and any change to a digital tool can be cumbersome. On the other hand, the prior investment of a professional needs to be considered. If professionals have spent large sums of money on analog assessment batteries, it is expected that they will be reluctant to change until the redemption of the investment is effective.

Besides, it should also be considered that access to the Internet is most of the time a mandatory technical requirement of the CCA. While there is usually a fast and stable Internet connection in urban spaces, there are places where the connection does not allow for assessments that require heavy or agile data transfer (Gerli and Whalley, 2021). The same happens with access to electricity, which is not always stable or possible, limiting access to this type of test. In addition, there is another critical aspect related to the specific testing conditions that must be taken into account when assessing remotely. Most tests are intended to be applied in laboratory-like clinical settings, with good control of potentially interfering environment-related factors (Robillard et al., 2018). When a person performs the assessment from home without being under the guidance of a professional, the chances increase that uncontrolled environmental conditions could interfere with the process (e.g., distractions, facilitations, or other elements that may affect the validity of the assessment). For this reason, a correct CCA necessarily implies advising about the necessity to prepare and secure the environment and conditions in which the assessment is to be performed and implementing mechanisms to assess the validity of the data.

In a broader sense, it should also be noted that not all tests need to be directly translated into a digital format, and that some if not all will require some adaptations or modifications. In these circumstances, it becomes especially important to ensure the psychometric qualities of CCA (Gates and Kochan, 2015) and to develop adequate quality assurance tests. Furthermore, all the modifications need to be endorsed by scientific evidence, similar to the procedure being currently developed to adapt classic laboratory 2D assessments to 3D virtual reality assessments (see Rocabado and Duñabeitia, 2022).

## 5. Discussion: implications of these changes

Health measures such as the lockdowns adopted during the COVID-19 pandemic have made the importance of CCA clear (Hsu et al., 2021). Nowadays digital tools are integrated into the daily life of professional and non-professional individuals at schools, healthcare centers, workplaces, and social contexts (Tomasik et al., 2020; Nordin et al., 2021; Paganin and Simbula, 2021). They are well-known and familiar tools for most users. The design and implementation of CCA based on these widespread digital tools could offer a better service to students, patients, or research participants, making the activities more attractive and dynamic, individualizing the tests, and updating the reference standards.

CCA is increasingly integrated into the daily lives of professionals and has a great and growing potential. Without sacrificing the quality of the tests, CCA allows for adapting assessment characteristics to the specific needs of the test-takers, increasing the accuracy of the measurements, assessing remotely on common devices available to a wide portion of society, and giving immediate and motivating feedback. However, CCA also has human limitations, such as users who are not proficient with digital tools or professionals who want to amortize the investment made in classic tests. Besides, certain environmental conditions may limit the degree of generalization of CCA, such as the need to have access to electricity and connectivity or the relative lack of control over the assessment environment. Thus, proper adaptations to digital format with good psychometric characteristics are essential to spread the use of CCA. Nevertheless, the transition to digital assessments is an inevitable evolution.

The debate is not about using only digitized tools or only classic tools, but about whether we are adapting classic paper-and-pencil tools to the context in which we live at the right speed. Classic assessment tools will and should continue being used for some time or, perhaps, always. But the migration to digital tools in parallel is indisputable, and it must be carried out thoroughly to facilitate professionals’ work in case we meet again in such adverse circumstances where in-person paper-and-pencil assessments cannot be used.

## Data availability statement

The original contributions presented in the study are included in the article/supplementary material, further inquiries can be directed to the corresponding author.

## Author contributions

DA and JD contributed to the conceptualization. DA wrote the original draft. JD reviewed, edited the text, and supervised the project. All authors contributed to the article and approved the submitted version.

## Funding

This research has been partially funded by grant PID2021-126884NB-I00 from the Spanish Government. The APC was funded by the publication fund of UiT, The Arctic University of Norway.

## Conflict of interest

The authors declare that the research was conducted in the absence of any commercial or financial relationships that could be construed as a potential conflict of interest.

## Publisher’s note

All claims expressed in this article are solely those of the authors and do not necessarily represent those of their affiliated organizations, or those of the publisher, the editors and the reviewers. Any product that may be evaluated in this article, or claim that may be made by its manufacturer, is not guaranteed or endorsed by the publisher.
